# A transgenic mouse model to inducibly target prosurvival Bcl2 proteins with selective BH3 peptides *in vivo*

**DOI:** 10.1038/cddis.2015.54

**Published:** 2015-03-12

**Authors:** E F Lee, M Takiguchi, A Pettikiriarachchi, M Evangelista, D C S Huang, R A Dickins, W D Fairlie

**Affiliations:** 1 Structural Biology Division, The Walter and Eliza Hall Institute of Medical Research, Parkville, Victoria, Australia; 2 Department of Medical Biology, The University of Melbourne, Parkville, Victoria, Australia

*Dear Editor*,

BH3-mimetic drugs that antagonize Bcl-2 family prosurvival proteins are effective against some cancers, particularly those with abnormally high expression of the prosurvival protein target.^1^ Despite the recent success of BH3-mimetics targeting Bcl-2, Bcl-x_L_ and Bcl-w, mechanism-based cell killing *in vivo* by targeting other important prosurvival proteins such as Mcl-1 or Bfl-1 has yet to be demonstrated. In the absence of small molecules targeting these prosurvival proteins, BH3-domain peptides, the prototypes for this class of drug, are useful because their binding specificity profile can be manipulated.^2, 3^ Such peptides have been applied in *in vitro* studies to validate the targeting of particular prosurvival proteins in certain tumor types.^4, 5^ However, *in vivo* applications require technically challenging chemical modifications of peptides, and while mouse xenograft models of virally-infected BH3 domain-expressing tumor cells can be used, this does not allow evaluation of effects of the ligand on normal tissues.

Here we have generated transgenic mice in which peptide-based BH3 ligands can be inducibly expressed to evaluate their effects *in vivo* and provide proof-of-principle for similar-acting drugs. To achieve this we adapted a previously described strategy allowing FLP-recombinase-mediated insertion of an expression cassette at an frt ‘landing pad' at the type I collagen (*Col1a1*) locus in mouse embryonic stem cells.^6^ The cassette comprises sequences encoding a BH3-domain protein under control of a tetracycline (tet)-regulated element (TRE) promoter. To develop a system that is broadly applicable to BH3 domains with different specificities, we employed the Bim_S_ BH3-only protein as a scaffold in which BH3 domains with different specificities could replace the native BH3 sequence (Figure 1a). Bim_S_ is an intrinsically unstructured protein that tolerates extensive mutation of its BH3 sequence, and Bim_S_BH3 chimeras display the prosurvival protein specificity profile of the replacement BH3 domain.^7^ In this study we focussed on the BH3 domain of Bad because it targets the prosurvival proteins Bcl-2, Bcl-x_L_ and Bcl-w.^7^ Hence Bim_S_Bad expression should mimic the BH3-mimetics ABT-737 and ABT-263 that have been extensively studied *in vivo*.^1^

We generated TRE-Bim_S_Bad transgenic mice and crossed them to mice expressing the rtTA (tet-on) transactivator under the control of the cytomegalovirus (CMV) promoter, which provides high-level expression in many tissues including blood.^8^ Bitransgenic mice were then treated with doxycycline (Dox) to induce Bim_S_Bad expression (Figure 1a). Western blot analysis verified Dox-inducible transgene expression in white blood cells of Bim_S_Bad; CMV-rtTA bitransgenic mice (Figure 1b). As ABT-737/263 induces thrombocytopenia in mice and humans due to antagonism of Bcl-x_L_, we measured platelet levels as a biomarker for functional expression of the ligand.^1^ Notably, blood cell analysis of these mice revealed a significant reduction (~65% decrease) in platelet counts (Figure 1c and d), a degree of thrombocytopenia comparable with that seen in patients administered with ABT-263.^9^ Importantly, platelet counts rebounded to normal levels following removal of Dox for 7 days (Figure 1d), illustrating the reversibility of the system. We also generated an additional transgenic mouse strain allowing inducible expression of a Bim_S_Bad construct possessing a BH3 sequence mutation (Bim_S_Bad^mut^; Figure 1a) that decreases its affinity for Bcl-x_L_ by >40-fold (K_D_ 8.5 nM *versus* <0.2 nM as measured by surface plasmon resonance). Induction of Bim_S_Bad^mut^ expression (Figure 1b) did not alter blood cell or platelet counts (Figure 1d), indicating that the thrombocytopenia observed in Bim_S_Bad mice is due to Bcl-x_L_ inhibition. These data provide the first evidence that BH3-only proteins can be inducibly expressed in a mouse model, effectively mimicking at least one functional consequence (reduced platelet counts) seen in mice and humans treated with BH3-mimetic drugs of similar specificity. We envisage multiple applications for similarly engineered mice. For example, tet-regulated expression of different Bim_S_ variants in mice could reveal toxicities associated with neutralization of their prosurvival protein targets (individually or in combination), and tissue-specific effects could be addressed by crossing onto mice where rtTA expression is driven by different promoters. Moreover, mice crossed to different tumor-prone models could provide *in vivo* evidence for the tumor-killing efficacy associated with different BH3 specificities not yet available through small molecule drugs, and extended to studies on combination therapies with existing drugs.

## Figures and Tables

**Figure 1:**
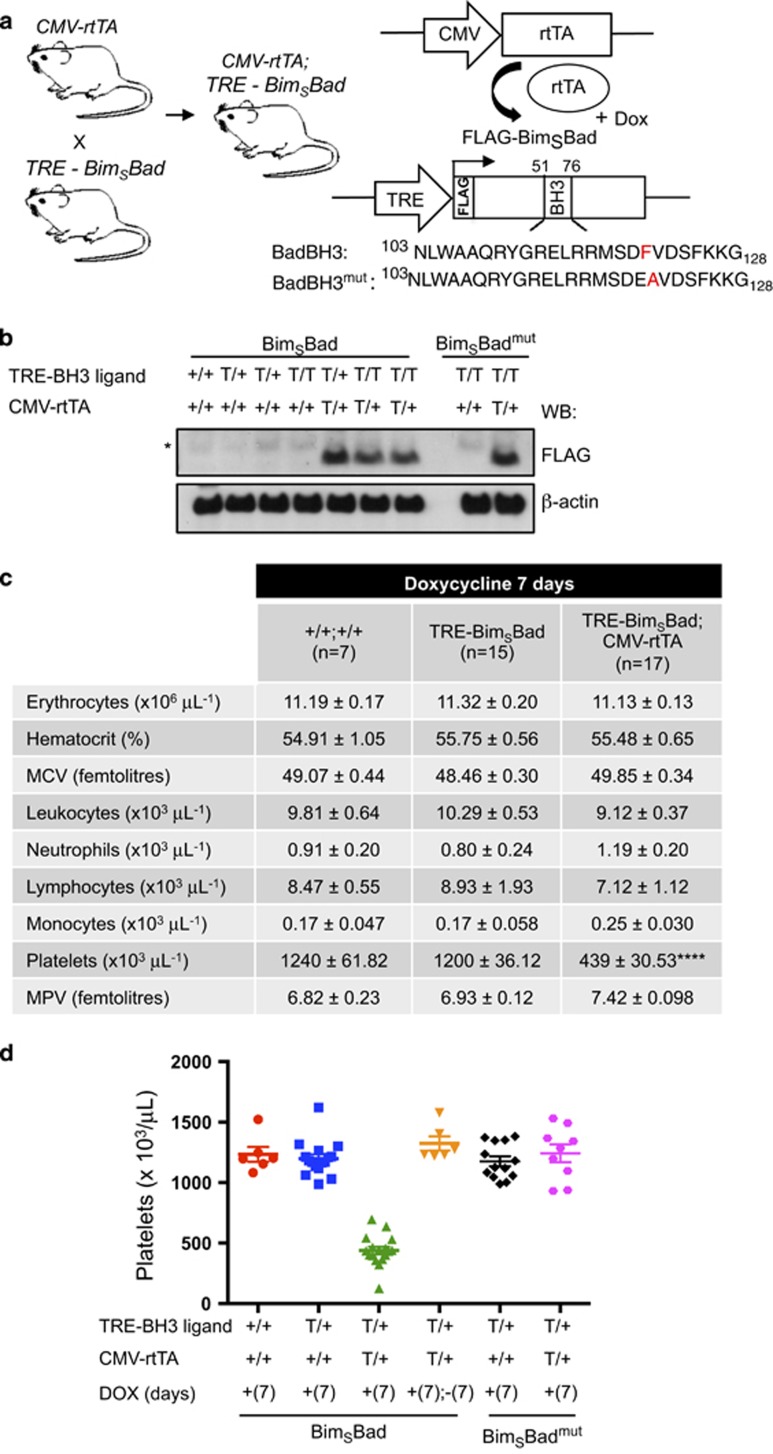
Expression of Bim_S_Bad reduces platelet levels in mice. (**a**) Schematic of the CMV-rtTA and TRE-Bim_S_Bad transgenes. Upon Dox treatment of bitransgenic mice, the rtTA (tet-on) protein transactivates the TRE promoter to drive expression of N-terminally FLAG tagged Bim_S_Bad. Targeting vectors were generated by cloning MluI-flanked FLAG-Bim_S_Bad/Bim_S_Bad^mut^ PCR amplicons into the MluI site of a modified version of the pgkATGfrt vector^6^ in which the TRE promoter has been replaced with TREtight. BH3 domain numbering refers to the amino acid residue position within the respective Bim_S_ or Bad protein sequences. The BadBH3 residue in red (F121) was mutated to alanine in the Bim_S_Bad^mut^ mice. Targeted ES cell clones were injected into C57Bl/6 blastocysts and chimeras crossed to C57Bl/6 female mice. All mouse colonies were maintained by C57Bl/6 backcrossing. (**b**) Western blot of white blood cells isolated from TRE-Bim_S_Bad/Bim_S_Bad^mut^; CMV-rtTA bitransgenic mice or wild-type or Bim_S_Bad/Bim_S_Bad^mut^ single transgenic control mice following 7 days of Dox food (600 mg/kg). Blood (100 *μ*l) was cleared of red blood cells by 10-fold dilution in red cell lysis buffer for 5 min followed by centrifugation. The white blood cell-containing pellet was then resuspended in lysis buffer (20 mM Tris pH 7.4, 135 mM NaCl, 1.5 mM MgCl_2_, 1 mM EGTA, 1% Triton X100 and 10% glycerol) for 1 h on ice, followed by centrifugation. The supernatant was then analyzed by Western blot probed with an anti-FLAG antibody and reprobed with anti-β-actin as loading control. * Indicates a non-specific band (**c**) Blood cell parameters after Dox treatment of bitransgenic and control animals for 7 days. ****P<0.0001 (*t*-test) compared with littermate controls (**d**) Platelet levels in TRE-Bim_S_Bad bitransgenic mice treated with Dox for 7 days rebound to normal levels after 7 days without Dox treatment. No changes in platelet levels were observed in Bim_S_Bad^mut^ mice following Dox treatment
